# Comprehensive analysis of PILRΑ’s association with the prognosis, tumor immune infiltration, and immunotherapy in pan-cancer

**DOI:** 10.1038/s41598-023-41649-6

**Published:** 2023-08-31

**Authors:** Qiao Li, Zhirong Yang, Xiaoyan He, Xin Yang

**Affiliations:** 1 Department of Pathology, People’s Hospital of Deyang City, Deyang, China; 2 Department of Surgery, People’s Hospital of Deyang City, Deyang, China

**Keywords:** Cancer, Computational biology and bioinformatics, Immunology, Biomarkers, Oncology

## Abstract

Paired immunoglobulin-like type 2 receptor alpha (PILRA) plays a vital role in regulating broad immune responses. However, the roles of PILRA in cancer immunity remain unexplored yet. In the current study, we comprehensively analyzed the oncogenic and immunologic roles of PILRA at a pan-cancer level based on the Cancer Genome Atlas and Gene Expression Omnibus datasets. PILRA was significantly dysregulated and frequently mutated in pan-cancer. Its expression and mutation status significantly impacted patient prognosis in several cancers. Besides, PILRA expression was positively correlated with ESTIMATE scores and the abundances of tumor-infiltrating immune cells. Concurrently, PILRA expression was significantly associated with predictive biomarkers of cancer immunotherapy, and positively correlated with the prognostic outcomes of cancer patients receiving immunotherapy. Mechanistically, enrichment analysis implied that PILRA might be involved in the regulation of immune response and metabolic process. This study uncovered the immunological roles of PILRA in cancers and its potential as a novel biomarker and therapeutic target for cancer immunotherapy.

## Introduction

Cancer remains to be an insurmountable challenge that threatens human life in spite of the rapid advancement of cancer therapies^1^. As compared to chemotherapy and targeted therapy to which drug resistance is inevitable, immunotherapy is emerging as a novel therapeutic approach with considerable potential in effectively relieving and even curing previously untreatable malignancies^2, 3^. However, a substantial proportion of cancer patients still respond poorly to immunotherapy^4, 5^. Of note, tumors undergo genetic alterations to elude the immune systems, largely undermining therapeutic efficacy^6^. Therefore, clarifying the molecular determinants that govern the immune evasion of cancers is important to optimize therapeutic outcomes.

The inhibitory receptor paired immunoglobulin-like type 2 receptor alpha (PILRΑ), along with the activating receptor PILRβ belongs to the PILR family^7^. Bearing two immunoreceptor tyrosine-based inhibition motifs (ITIMs) in its cytoplasmic domain, PILRΑ functions to transmit inhibitory signals by recruiting SHP-1 and SHP-2, upon recognition of ligands such as CD99, neuronal differentiation proliferation factor-1 and CD8A, etc^8, 9^. PILRΑ, which is mainly expressed on immune cells, including macrophages, dendritic cells and granulocytes, is related to regulation of broad immune responses^10, 11^. For example, it has been reported that PILRΑ negatively regulates neutrophil infiltration via modulation of integrin activation during inflammation^12^. Besides, PILRA controls monocyte mobility through regulating integrin signaling and inhibiting CD99–CD99 binding^13^. Furthermore, Zheng L and colleagues lately found that PILRΑ could interact with CD8A and keep CD8 T cell in a quiescent state, thus maintaining the T cell pool size in the absence of antigen exposure^14^. However, the involvement of PILRΑ in anticancer immunity remains poorly understood. Thus, elucidation of the immunologic roles of PILRΑ in cancers might offer a novel perspective for cancer immunotherapy.

In the present study, we sought to explore the expression profiles, and the prognostic and diagnostic value of PILRΑ in pan-cancer by analyzing the multi-omics data sourced from multiple online databases, and immunohistochemistry analysis was performed to verify its expression status in non-small cell lung cancer (NSCLC). Furthermore, we conducted an integrative bioinformatics analysis on the association of PILRΑ with tumor immune landscape and tumor immunotherapy. Finally, we investigated the functional mechanisms underlying the oncogenic and immunologic roles of PILRΑ across various cancers. Our study offers a comprehensive insight into the prognostic and immunological roles of PILRA across various cancers, and might facilitate the discovery of novel biomarkers and genetic drivers regarding cancer immunity.

## Results

### The aberrant expression profiles of PILRΑ in pan-cancer

**Figure 1:**
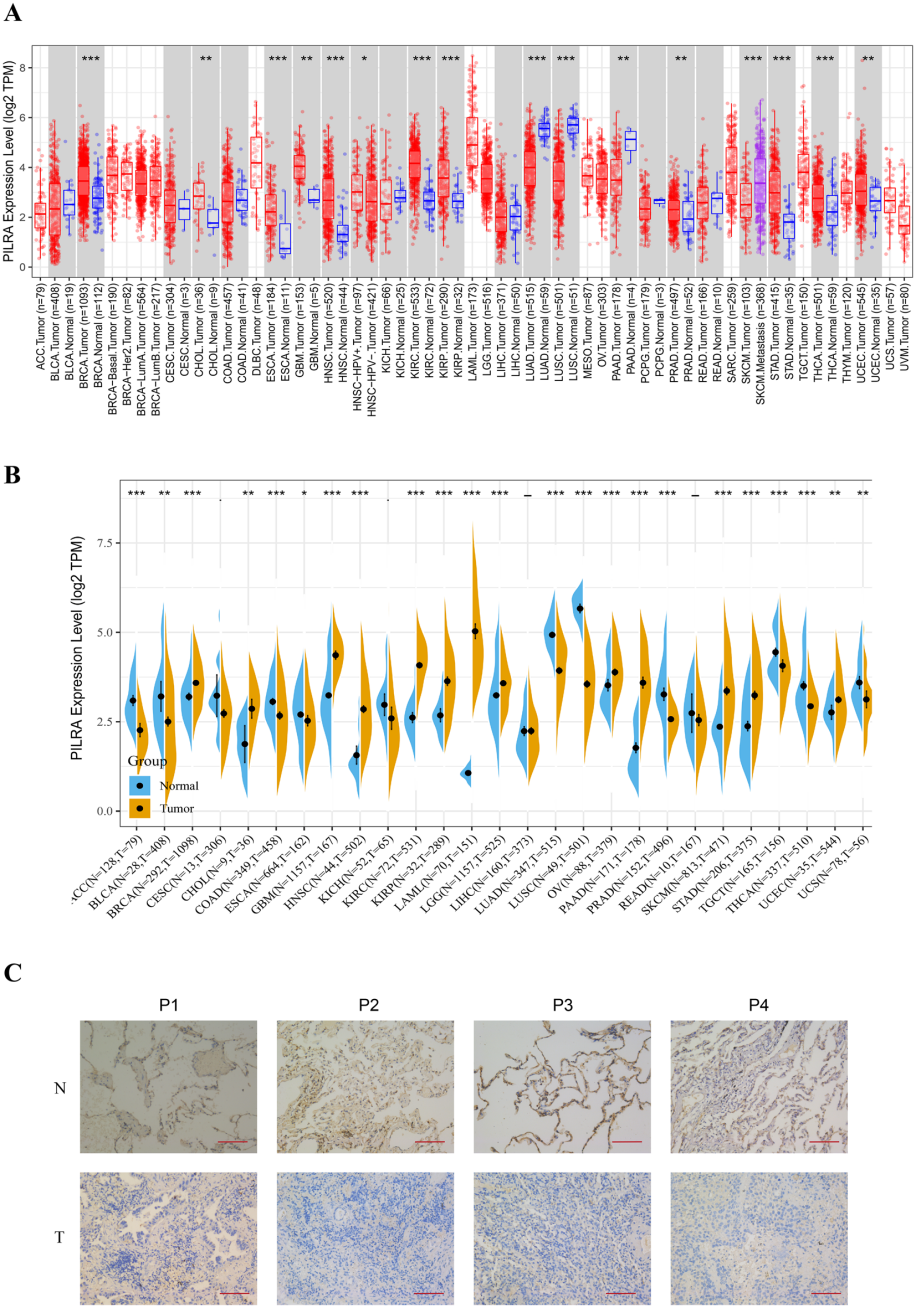
Analyses on the differential expression of PILRΑ in pan-cancer. **(A)** The differences of PILRΑ mRNA expression between tumor and normal tissues in 33 cancer types were analyzed by TIMER2.0 online tool based on TCGA datasets. Distributions of gene expression levels were displayed using box plots. Red and blue color represent cancerous and normal tissues, respectively. SKCM metastasis tissues are marked in purple. **(B)** The bean plots were plotted to compare PILRΑ expression in tumor tissues from TCGA datasets with that in normal tissues from GTEx datasets. **(C)** PILRA protein levels in tumor and corresponding normal tissues of NSCLC patients were evaluated by IHC (Scale bars, 100 μm). *, **, and *** correspond to *P* < 0.05, *P* < 0.01, and *P* < 0.001, respectively. *P* < 0.05 was considered significant.

To evaluate the differential expression of PILRΑ between tumorous and normal tissues, we investigated the expression level of PILRΑ mRNA using TIMER2.0 database. According to the results, PILRΑ expression was significantly upregulated in breast invasive carcinoma (BRCA), cholangiocarcinoma (CHOL), esophageal carcinoma (ESCA), glioblastoma multiforme (GBM), head and neck squamous cell carcinoma (HNSC), kidney renal clear cell carcinoma (KIRC), kidney renal papillary cell carcinoma (KIRP), stomach adenocarcinoma (STAD), thyroid carcinoma (THCA) and uterine corpus endometrial carcinoma (UCEC) while downregulated in only three types of cancer including lung adenocarcinoma (LUAD), lung squamous cell carcinoma (LUSC) and pancreatic adenocarcinoma (PAAD) as opposed to their normal tissues (Fig. 1A). Of note, the number of TCGA normal samples was relatively small for convincing statistical analysis, and several cancers are even lack of matched normal tissues. Therefore, we performed a complementary analysis by comparing the expression level of PILRΑ in tumor tissues with that in the GTEx normal tissues. According to the results, PILRΑ was differentially expressed in most of the cancers analyzed (Fig. 1B). Besides, we further confirmed that the PILRΑ protein expression was significantly elevated in breast cancer, PAAD, KIRC, and GBM, while decreased in LUAD and hepatic cell carcinoma (HCC) as compared to their corresponding normal tissues (see Supplementary Fig. S1 online). To verify the decreased expression of PILRA in NSCLC, immunohistochemistry (IHC) staining was performed in tumor and adjacent normal tissues, and the results confirmed that PILRA was significantly downregulated in NSCLC (Fig. 1C). Collectively, these data imply that PILRΑ gene is differentially expressed in multiple cancers as compared to normal counterparts.

### Correlation of PILRΑ expression with patients’ prognosis and diagnosis

**Figure 2:**
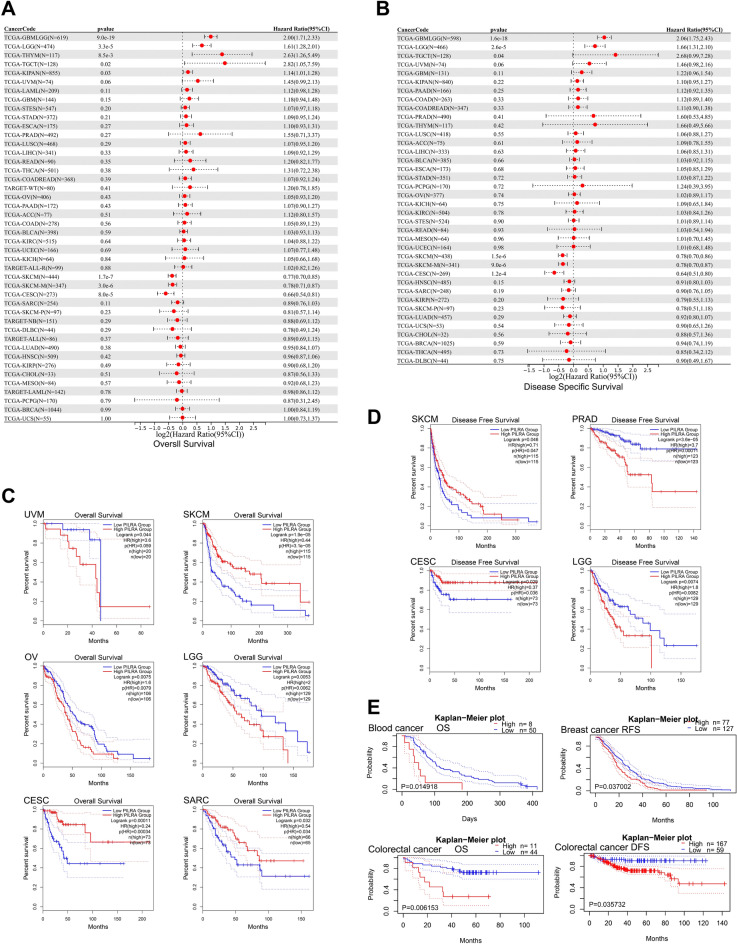
Survival analysis according to the high and low expression of PILRΑ in pan-cancer. **(A,B)** The forest plots showing the relevance of PILRΑ expression with the overall survival (**A**) and disease specific survival (**B**) of different cancers in the TCGA cohort. **(C,D)** Kaplan-Meir curves for the prediction of patients’ overall survival of UVM, SKCM, OV, LGG, CESC and SARC (**C**), and disease-free survival of SKCM, PRAD, CESC and LGG (**D**) based on PILRΑ expression via GEIPIA2.0. **(E)** Kaplan-Meir plots showing the association between PILRA expression and the survival of Blood, breast and colorectal cancer based on PILRΑ expression using Prognoscan websites. *P* < 0.05 was considered statistically significant.

To evaluate the prognostic value of PILRΑ in pan-cancer, the relationship between PILRΑ expression and patients’ clinical outcomes was assessed by performing univariate cox regression analysis of TCGA data. As shown in Fig. 2A, B, high expression of PILRΑ was inversely correlated with both overall survival (OS) and disease specific survival (DSS) in glioma (GBMLGG), brain lower grade glioma (LGG) and testicular germ cell tumors (TGCT) whereas significantly positive correlation was observed in skin cutaneous melanoma (SKCM) and cervical squamous cell carcinoma and endocervical adenocarcinoma (CESC). Besides, we employed GEPIA2.0 for Kaplan–Meier survival analysis, the results of which showed that PILRΑ expression was significantly associated with poorer OS in patients with uveal melanoma (UVM), ovarian serous cystadenocarcinoma (OV) and LGG while correlated with significantly prolonged survival in SKCM, sarcoma (SARC) and CESC (Fig. 2C). Moreover, the results of Kaplan–Meier survival analysis further demonstrated that PILRΑ expression was in significantly negative correlation with DFS in patients with PRAD and LGG whereas positive association of PILRΑ expression with DFS was identified in patients with CESC and SKCM (Fig. 2D). Finally, the relationship between PILRΑ expression and patients’ prognosis in each cancer type was further analyzed using Prognoscan website. According to the results, PLIRA expression level was negatively associated with patients’ prognosis in three types of cancer, including: blood cancer (OS: *p* = 0.014918), colorectal cancer (OS: *p* = 0.006153; DFS: *p* = 0.035732) and breast cancer (RFS: *p* = 0.037002) (Fig. 2E).

**Figure 3:**
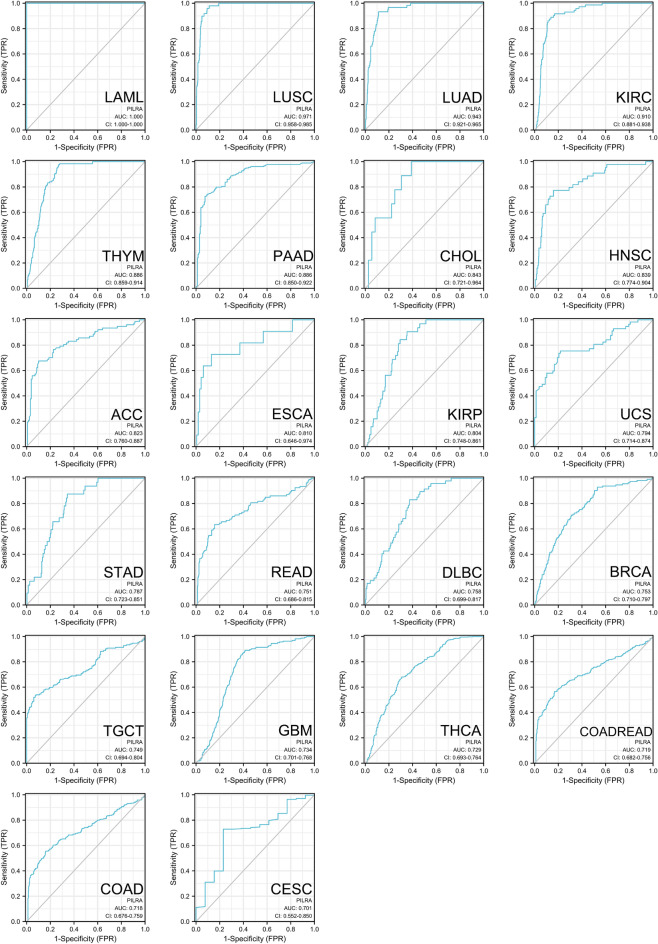
ROC curve analysis evaluating the diagnostic potency of PILRΑ in pan-cancer based on TCGA datasets. 22 cancers with AUC > 0.7 for PILRA were displayed here.

Next, the diagnostic value of PILRΑ in pan-cancer was explored through analysis of TCGA dataset. As visualized by the ROC curves, PILRΑ expression levels exhibited strong diagnostic potency (AUC > 0.9) in LAML, LUSC, LUAD and KIRC, and moderate diagnostic potency (0.9 > AUC > 0.7) in 18 types of cancer, including: THYM, PAAD, CHOL, HNSC, adrenocortical carcinoma (ACC), ESCA, KIRP, uterine carcinosarcoma (UCS), STAD, rectal cancer (READ), DLBC, BRCA, TGCT, GBM, THCA, colon adenocarcinoma/rectum adenocarcinoma esophageal carcinoma (COADREAD), colon adenocarcinoma (COAD) and CESC (Fig. 3). The ROC curves for the remaining 8 cancers for which PILRA only exhibited a weak diagnostic potential (AUC < 0.7) can be found as Supplementary Fig. S2 online. Taken together, these data indicate that PILRΑ is a potential diagnostic factor in various cancers.

### Genetic alteration and genome-wide association of PILRΑ in pan-cancer

**Figure 4:**
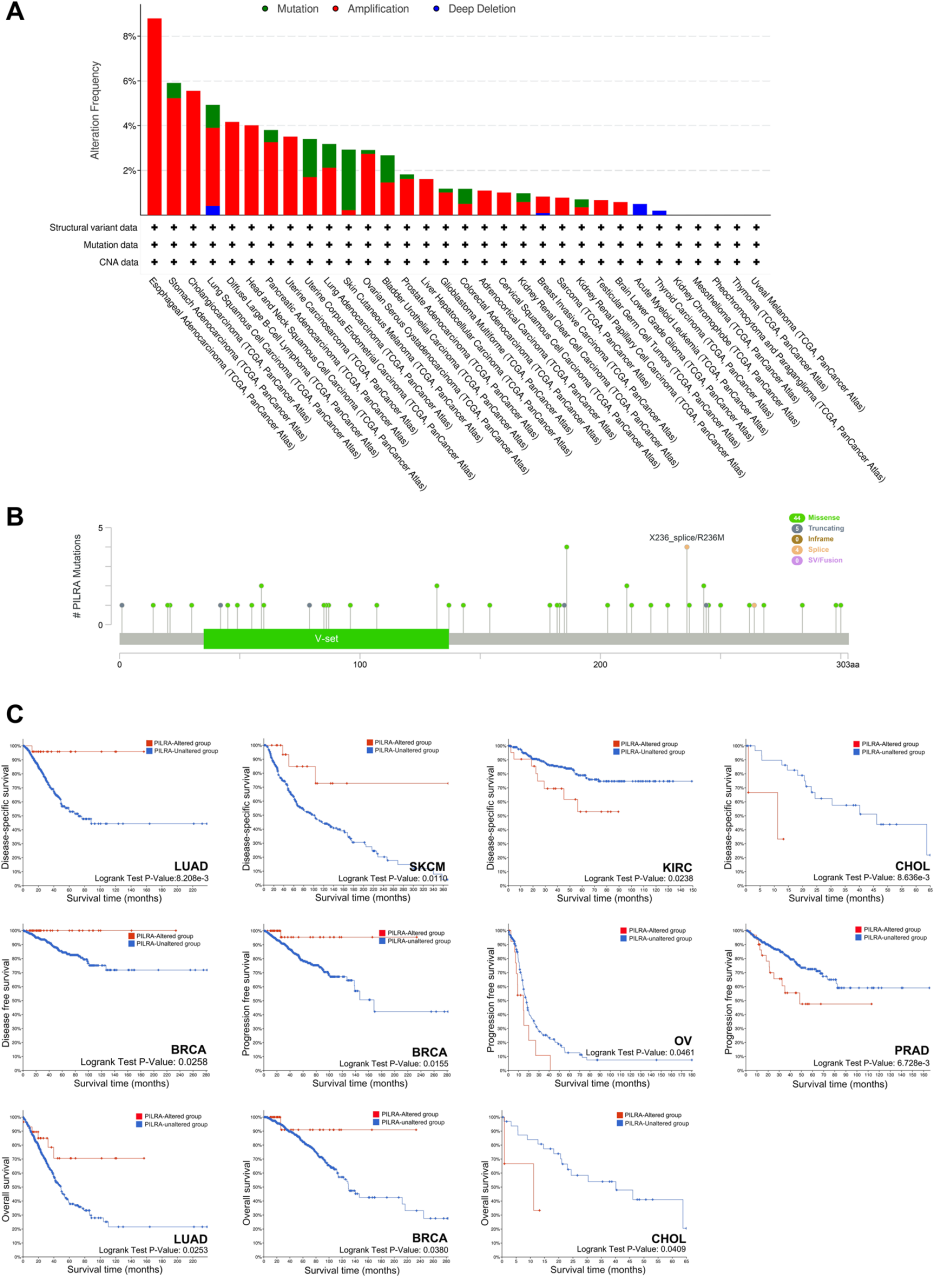
The mutation landscape for PILRΑ. **(A)** Bar charts showing the genetic alteration frequency of PILRΑ across different cancers based on cBioPortal website. Mutation, amplification and deep deletion were included as the genetic alteration. **(B)** The mutation sites spanning the protein domains of PILRΑ, containing Missense, Truncating, Inframe, Splice and SV/Fusion. **(C)** Associations of PILRA genetic alterations with prognosis in pan-cancer.

Cancers feature a stark rise in genomic alterations across the genome^15^. In order to inspect the PILRΑ mutation profiles in different cancers, three main genetic alteration categories of PILRΑ, including amplification, deep deletion and mutation, was assessed based on analysis of TCGA datasets using cBioPortal. As shown in Fig. 4A, the alteration frequency of PILRΑ was the highest in esophageal adenocarcinoma (> 8%), followed by STAD, CHOL, LUSC and DLBC. Notably, the most common alteration type of PILRΑ was “Amplification”, of which the frequency markedly exceeded that of “Mutation” and “Deep deletion” in various cancers (Fig. 4A). Besides, as illustrated by the mutation diagram, a total of 53 mutation sites were identified in PILRΑ, among which R236M was the dominant mutation spot (Fig. 4B). Genetic alterations might be associated with patients’ clinical survival outcomes in various cancer types^16^. Therefore, we compared survival differences between PILRΑ-altered and -unaltered groups in pan-cancer using cBioPortal, and found that the presence of PILRΑ alterations was associated with better prognosis in LUAD, SKCM and BRCA, while inversely related to patients’ survival in KIRC, CHOL, OV and PRAD (Fig. 4C).

**Figure 5:**
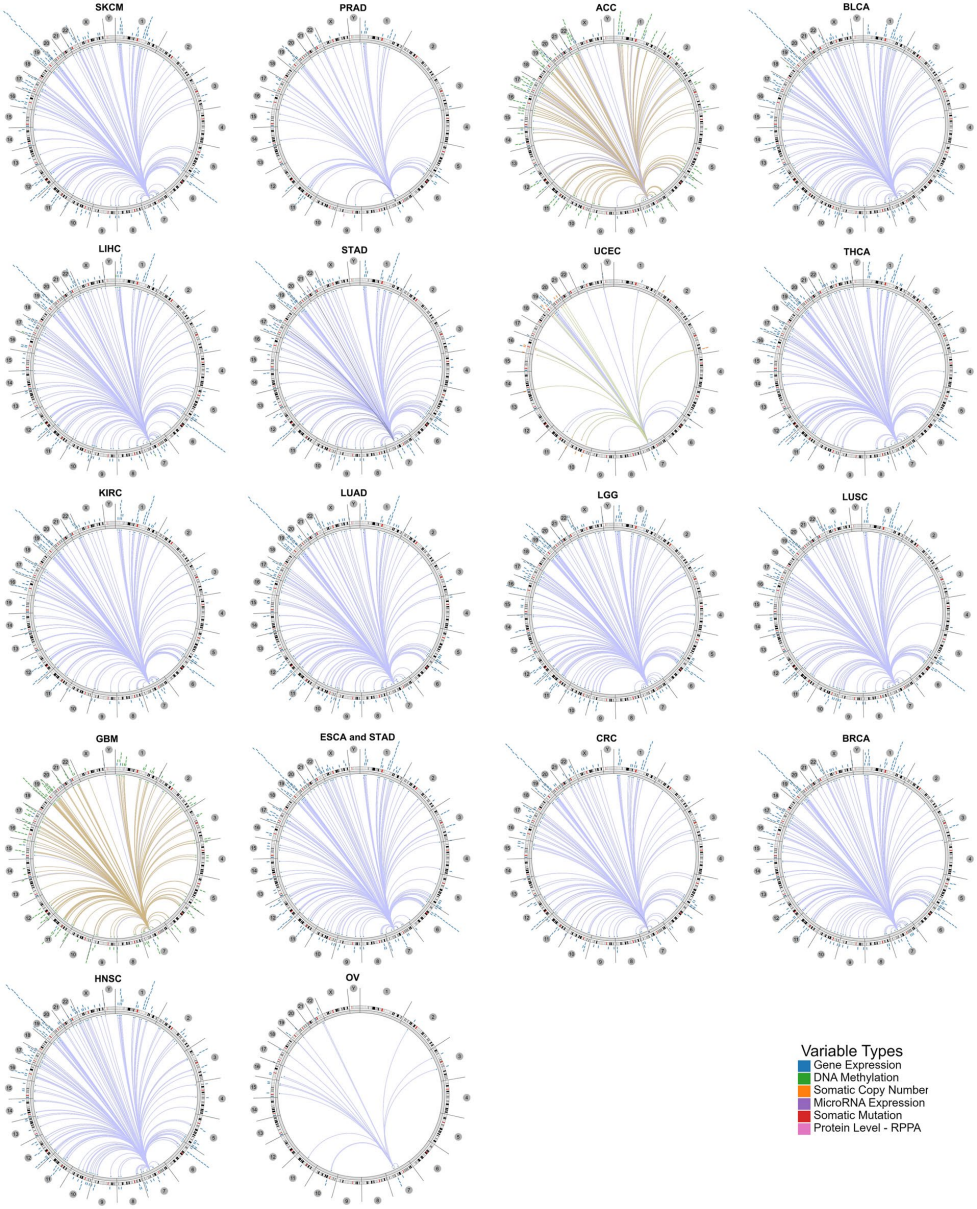
Genome level view of the association between PILRΑ and other molecular signatures within the context of genomic coordinates using Cancer Regulome website.

Additionally, we analyzed the genome-wide associations of PILRΑ expression with several molecular features, including gene expression, DNA methylation, somatic copy number, microRNA expression, somatic mutation and protein level-RPPA on Cancer Regulome Tools. As illustrated by circus plots, associations between PILRΑ and other molecular signatures were observed and visualized according to genomic coordinates in SKCM, PRAD, ACC, BLAC, LIHC, STAD, UCEC, THCA, KIRC, LUAD, LGG, LUSC, GBM, ESCA and STAD, CRC, BRCA, HNSC and OV **(**Fig. 5**)**.

### The relationship between PILRΑ expression and tumor immune infiltration in cancers

**Figure 6:**
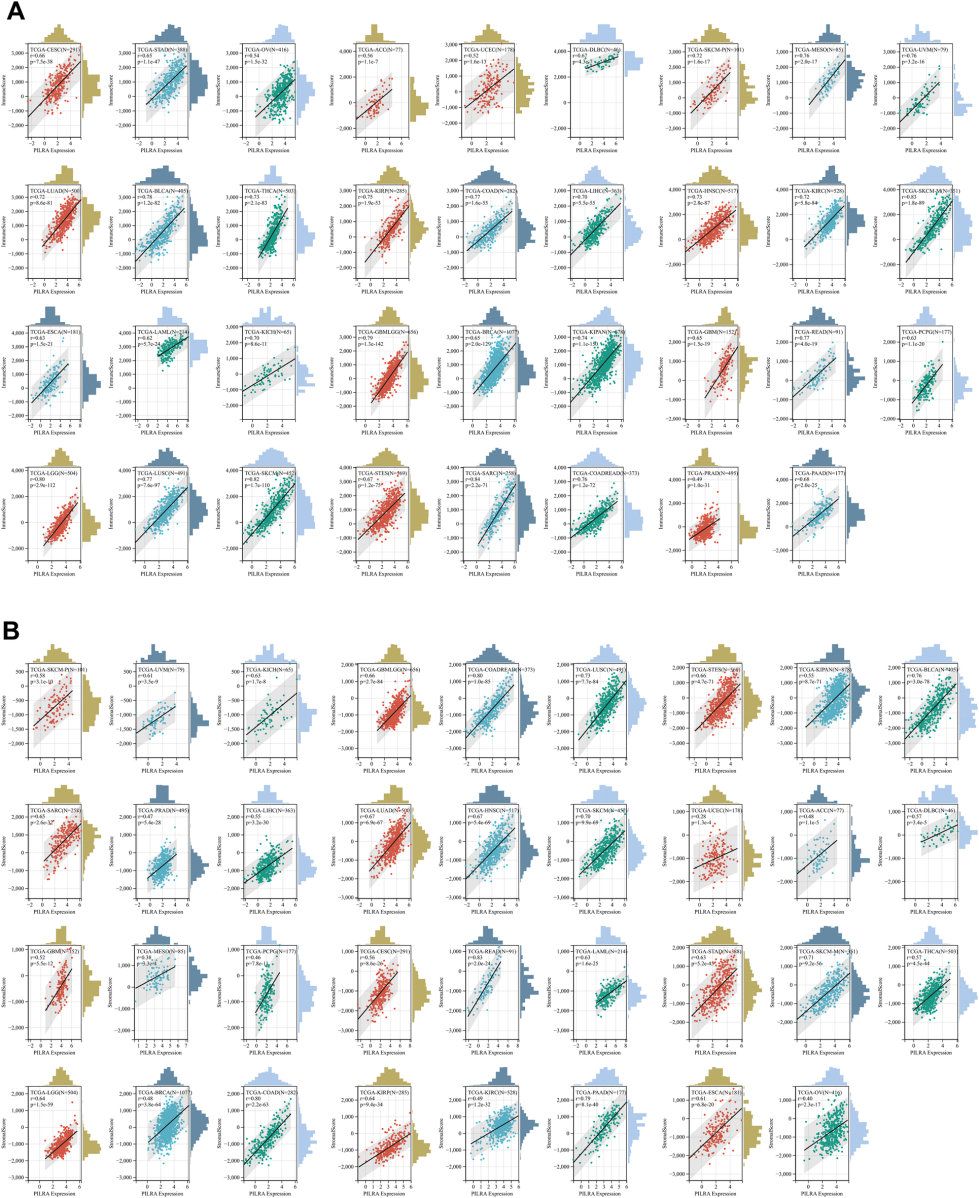
Associations of PILRA to immune score and stromal score. **(A,B)** Scatter plots showing the correlation between PILRΑ and immune score (**A**), and stromal score (**B**) in various cancers. *P* < 0.05 was considered significant.

**Figure 7:**
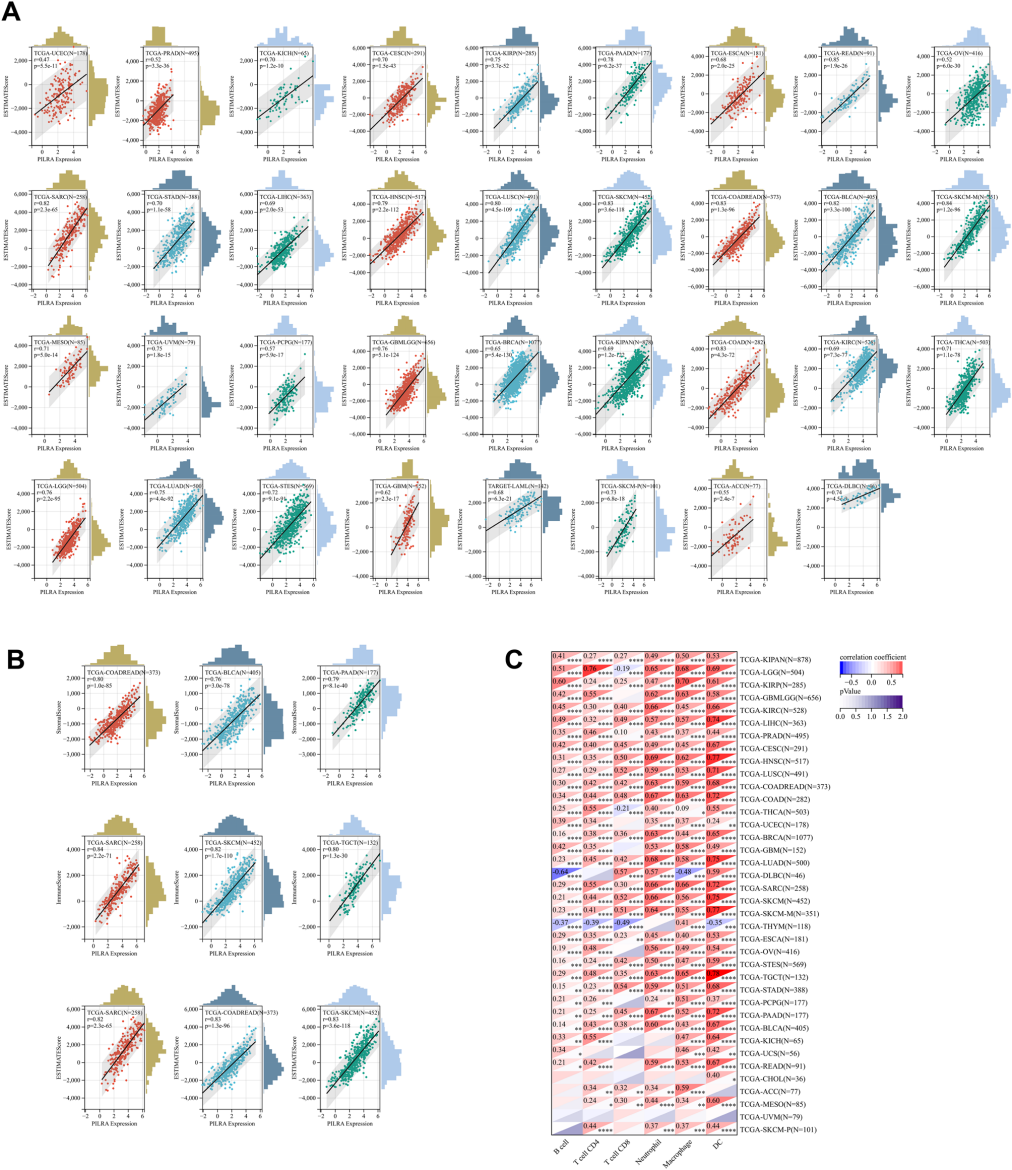
Associations between PILRΑ and immune infiltration. **(A)** The correlation between PILRΑ expression and ESTIMATE score in pan-cancer. **(B)** Scatter plots displaying the correlation of PILRΑ with Immune score, Stromal score and ESTIMATE score in the top three cancers. **(C)** The heatmap showing the relation between PILRΑ expression and levels of six tumor-infiltrating immune subsets in pan-cancer using TIMER. *P* < 0.05, **P < 0.01, ***P < 0.0011, ****P < 0.0001. *P* < 0.05 was considered significant.

Cancer immunotherapy largely depends on the accumulation and activity of immune effector cells within the TME, since increased infiltration of immune cells into tumors is associated with an immuno-supportive TME^17^. To understand the immunological roles of PILRΑ within the cancer microenvironment, we employed “estimate” R package to estimate the abundance of stromal cells and immune cells based on the PILRΑ expression in a total of 10,180 tumor samples. According to the results, PILRΑ expression showed a positive correlation with immune score (Fig. 6A), stromal score (Fig. 6B) and ESTIMATE score (Fig. 7A) in 35 types of cancer in a consistent manner. Notably, the strongest positive association of PILRΑ expression with immune infiltration was observed in several cancers including SARC, SKCM and COADREAD (Fig. 7B). Additionally, we evaluated the relationship between PILRΑ expression and infiltration scores of six main immune cell subtypes (including B cell, CD4 T cell, CD8 T cell, Neutrophil, Macrophage, dendritic cell) via TIMER2.0. As depicted in Fig. 7C, PILRΑ expression significantly correlated with immune infiltration in various cancers (Fig. 7C). Thus, these data indicate that PILRΑ is putatively implicated in tumor immune infiltration.

### Analysis of the relationship between PILRΑ expression and response to immunotherapy in pan-cancer

**Figure 8:**
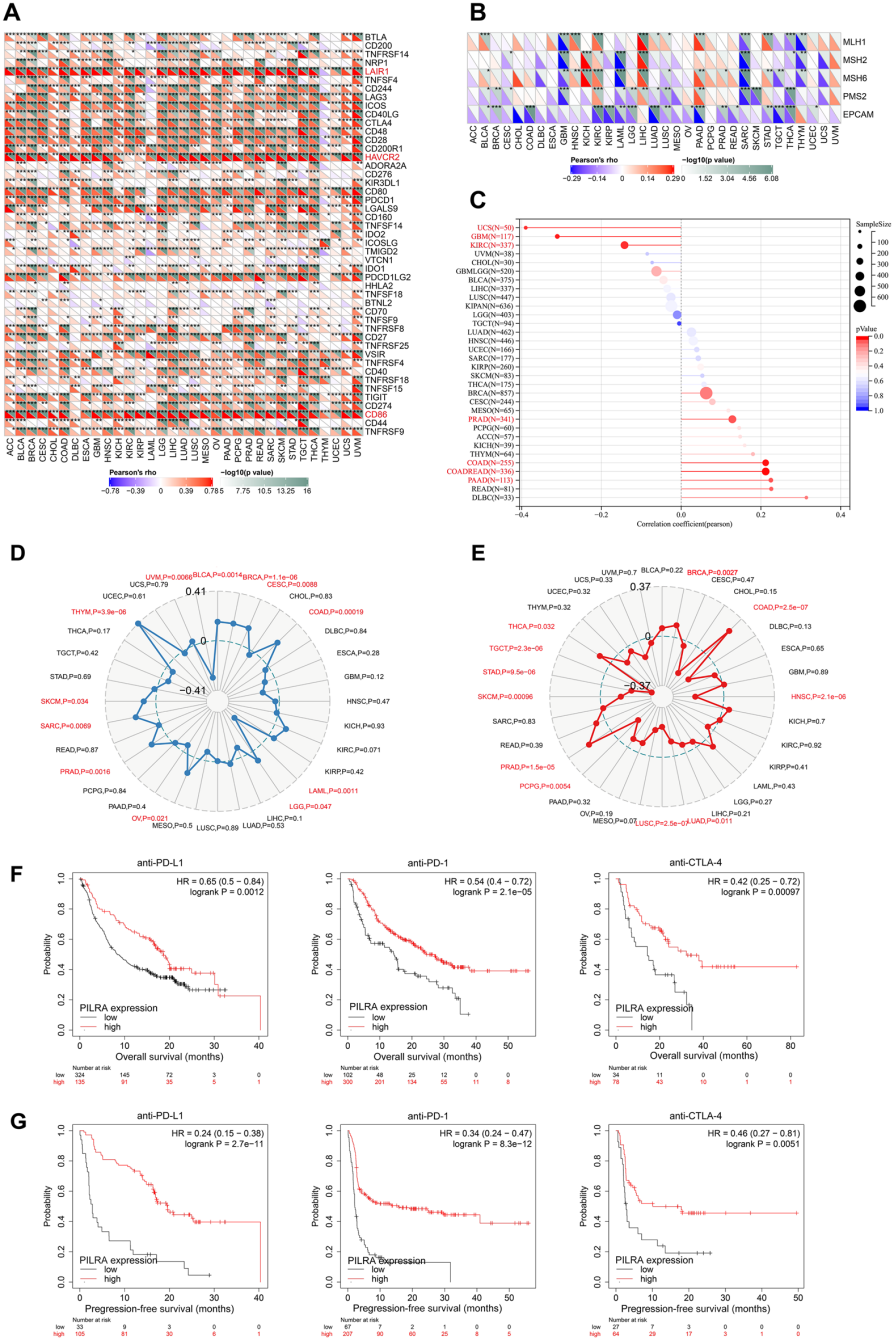
Associations of PILRΑ expression with immunity-associated markers in pan-cancer. **(A)** The heatmap exhibiting the correlation between PILRΑ and levels of 60 immune checkpoint genes (ICGs) in pan-cancer. ICGs with a close and positive correlation with PILRA expression were labeled in red. **(B)** The correlations between PILRΑ and levels of 5 genes that are key to intact MMR functions in multiple cancers. **(C)** The bar chart displaying the correlation coefficients between neoantigen levels and PILRΑ. Red color indicated statistical significance. **(D,E)** The radar charts display the correlations between TMB (**D**) and MSI (**E**), and PILRA in cancers, respectively. The dotted-line circles indicate correlation coefficients, the number of which were shown on the graphs. Red color indicated statistical significance. **(F,G)** Kaplan-Meir plots predicting patients’ overall survival **(F)** and progression-free survival **(G)** in cancers based on PILRΑ expression in Kaplan–Meier website. *P < 0.05, **P < 0.01, ***P < 0.001. *P* < 0.05 was considered significant.

The genetic landscape of tumors has been shown to be robust indicators of tumor immunity and might be used as predictors of immunotherapy^18^. Therefore, we sought to explore the correlation of PILRΑ expression with immune check point genes (ICGs), mismatch repair (MMR)-related genes, neoantigen load, and genomic instability as defined by TMB and MSI status, all of which are important predictive biomarkers of the efficacy of immunotherapies across various cancers. As shown in Fig. 8A, PILRΑ expression had a close and positive correlation with three ICGs including LAIR1, HAVCR2 and CD86 in almost all cancer types. Notably, PILRΑ expression was significantly associated with key members of the MMR system (EPCAM, MLH1, MSH2, MSH6, and PMS2) (Fig. 8B). With respect to neoantigen, PILRΑ was in significantly positive correlation with it in two types of cancer, including COAD and COADREAD while negatively correlated to it in three types of cancer, including GBM, KIRC and UCS (Fig. 8C). In addition, positive correlation between PILRΑ expression and TMB was observed in 10 types of cancer (including UVM, BRCA, CESC, COAD, LAML, OV, PAAD, SARC, SKCM and THYM) while a negative correlation was observed in only 2 types of cancer (including UVM and LAML) (Fig. 8D). Furthermore, PILRΑ expression was significantly associated with MSI in BRCA, COAD, HNSC, LUAD, LUSC, PCPG, PRAD, SKCM, STAD, TGCT and THCA (Fig. 8E). According to Kaplan–Meier plotter website, PILRA expression was positively associated with prognosis of cancer patients receiving immunotherapy, including anti-PD1, anti-PD-L1 and anti-CTLA-4 (Fig. 8F-G). Collectively, these results indicate that PILRΑ is a potential predictive biomarker of patients’ response to immunotherapy.

### Analysis of PILRΑ’s correlation with RNA modification and DNA methylation in pan-cancer

**Figure 9:**
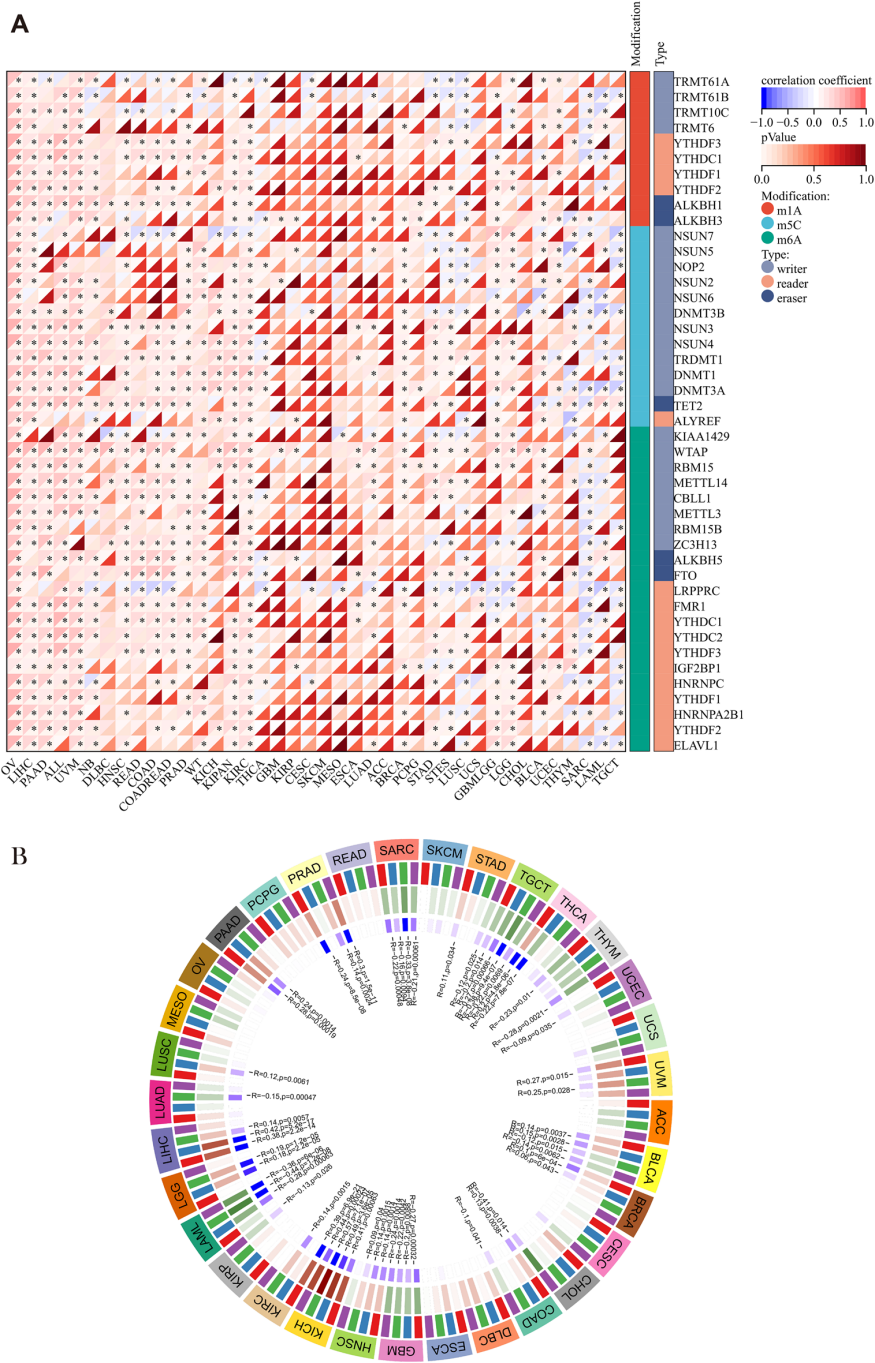
PILRΑ was implicated in epigenetic modulations in various cancers. **(A)** The heatmap exhibiting the associations of PILRΑ with 44 RNA-modulating genes in pan-cancer. The left and right bar indicates m^6^ARNA modification and modification enzyme types, respectively. *P < 0.05. **(B)** The circus plot showing the correlations between PILRΑ expression and four methyltransferases (DNMT1, DNMT2, DNMT3A and DNMT3B), with darker shades of blue blocks in innermost layer corresponding to lower *P* value. The brown- and green-colored blocks in the second layer corresponds to positive and negative correlation coefficient values, respectively. In the third layer, DNMT1, DNMT2, DNMT3A and DNMT3B were shown in red, blue, green and purple, respectively. The outmost layer indicates cancer names. *P* < 0.05 was considered significant.

Accumulating evidence demonstrates that tumors commonly hijack epigenetic mechanisms, such as RNA modification and DNA methylation, to regulate immunity, thus evading immune suppression^19–21^. To explore the relevance between PILRΑ and epigenetic modulations in pan-cancer, we analyzed the correlation of PILRΑ with 44 genes categorized into three broad types of RNA modification (m1A, m5C, m6A). As depicted in Fig. 9A, PILRΑ had positive correlations with a majority of RNA modulator genes in most of the cancers. Additionally, we investigated PILRΑ’s correlation with DNA methylation status, and a significant association was observed between PILRΑ and four DNA methyltransferases in 23 cancers (Fig. 9B). Notably, the highest co-expression coefficients (> 0.4) were observed between PILRA expression and four methylation transferases in KICH.

### Enrichment analysis of PILRΑ-related pathways in pan-cancer

**Figure 10:**
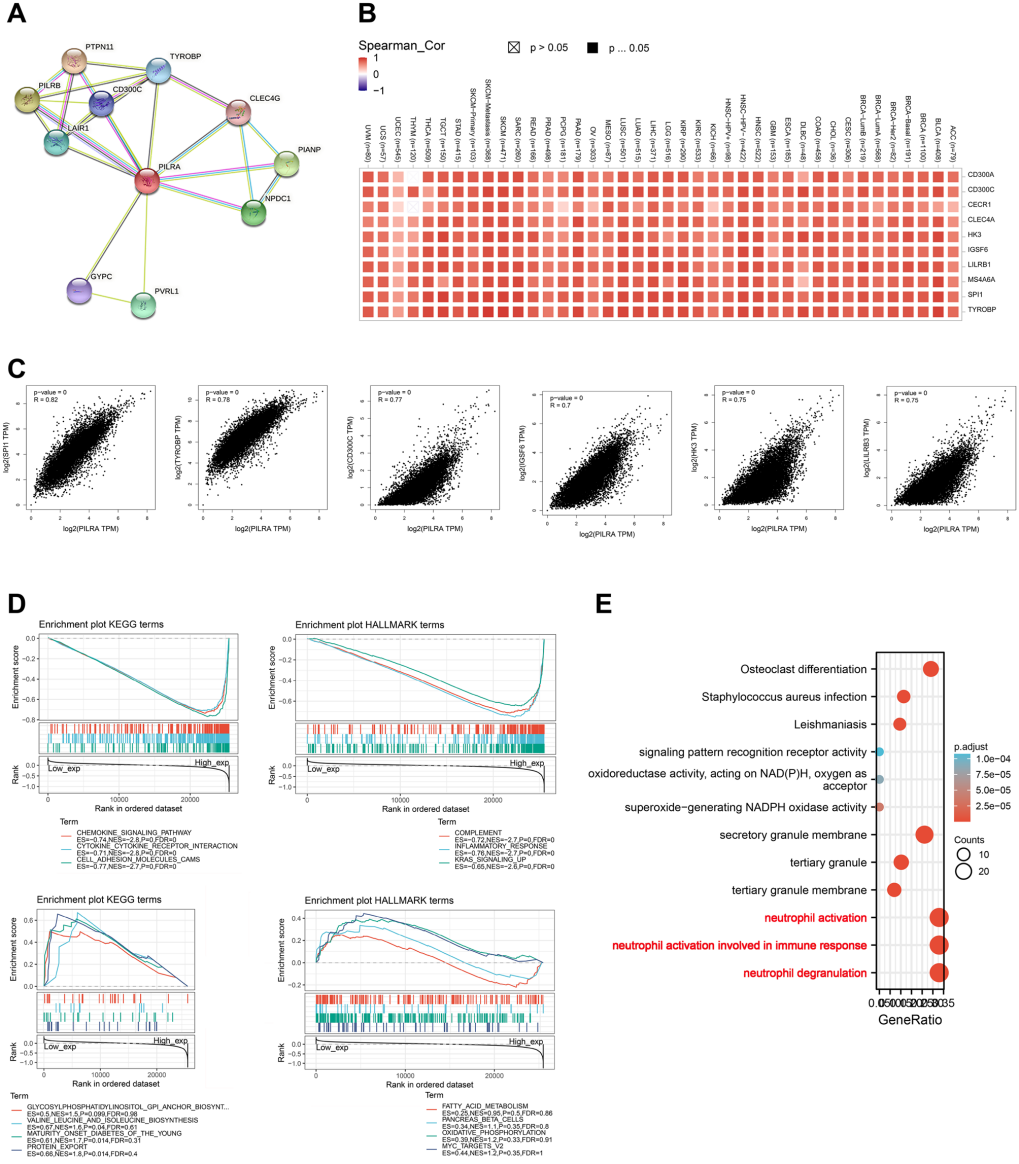
The function profiles of PILRΑ in pan-cancer. **(A)** The network of experimentally validated PILRΑ-interacting partners visualized via STRING web tool. **(B,C)** The heatmap (**B**) and scatter plots (**C**) showing the correlations between PILRΑ and its top 5 related genes in pan-cancer using GEPIA2.0. Spearman_Cor is short for Spearman correlation. **(D)** GSEA analysis of the enriched KEGG and HALLMARK terms according to the expression of PILRΑ in pan-cancer. Top three mostly significantly enriched pathways were visualized. **(E)** GO and KEGG pathway analysis of PILRΑ-related functions in pan-cancer. *P* < 0.05 was considered significant.

To comprehend the functional roles of PILRΑ in cancers, interacting proteins and most relevant genes of PILRΑ were identified for conduction of functional enrichment analysis. The experimentally validated PPI network of PILRΑ was visualized using STRING databases (Fig. 10A). Besides, the top 100 co-expressed genes of PILRΑ in various cancers were displayed by the correlation heatmap on GEPIA2.0 (Fig. 10B). And the strongest correlation of PILRΑ was noticed with 6 genes, including SPI1, TYROBP, CD300C, IGSF6, HK3 and LILRB3) (Fig. 10C). According to the GSEA results of GO and KEGG, immune response and metabolic process were enriched for PILRΑ (Fig. 10D). In addition, the functional enrichment of GO terms indicated that PILRΑ was primarily associated with neutrophil activation, neutrophil activation involved in immune response and neutrophil degranulation (Fig. 10E).

## Discussion

Recent therapeutic advancement in cancer immunotherapy has conferred a remarkable survival benefit to terminal cancer patients. However, it remains to be identified regarding a novel genetic alteration that encompasses a broader spectrum of patients for cancer immunotherapy. Herein, by performing integrative bioinformatics analysis, we report a novel tumor immune-associated gene, termed as PILRΑ, that is aberrantly expressed and significantly relates to patients’ prognosis in pan-cancer. Specifically, we unveil that PILRΑ is positively associated with tumor immune infiltration, and analyze the correlation between PILRΑ expression and tumor immune markers and immunotherapy. Moreover, we investigated the biological functions of PILRΑ in different cancers by performing pathway enrichment using GSEA, GO and KEGG analysis for the first time.

Cancer is a genetic disease arising from the accumulation of alterations in genes that participate in various oncogenic processes^22^. For example, combined Dusp4 and p53 loss with Dbf4 amplification drives tumorigenesis in breast cancer^23^. Neurofibromatosis 1 (NF1) mutation drives initiation of optic glioma through mediating dysregulation of neuronal activity and stimulating of optic nerve activity^24^. In the present study, we demonstrated that PILRΑ is abnormally expressed and associated with patients’ survival across different cancers. In addition, we uncovered that PILRΑ was highly mutated in several tumors, including EA, STAD, CHOL, LUSC and DLBC. By conducting survival analysis, we unprecedentedly illustrated that genetic alteration in PILRΑ was associated with improved patients’ survival in LUAD, BRCA and SCKM, while significantly shortened the survival in KIRC, CHOL, OV and PRAD patients. Taken together, these results uncover that both the expression and genetic alterations of PILRΑ correlates with patients’ prognosis in various cancers.

Our current work provides the first evidence of the immunological roles of PILRΑ in pan-cancer. Initially, PILRΑ was mainly recognized for its regulatory role in immune systems. For instance, PILRΑ was reported to be involved in the regulation of innate immunity through recognition of a CD99-like ligand^25^. PILRA plays an important role in HSV-1 infection of monocytes as a coreceptor that associates with gB^26^. Moreover, PILRA G78R variant was associated with significantly decreased levels of HSV-1 infection in macrophages^27^. However, the roles of PILRΑ in tumor-related immunity remain unexplored yet. Here, we uncover that PILRΑ is positively associated with tumor immune infiltration in most of the cancers analyzed. Additionally, we demonstrate here that the presence of TP53 mutations in PILRΑ high-expression group is more frequent than low ones in KICH, LGG, ACC, UCEC, PRAD, BLCA, BRCA, LIHC, PAAD, LUAD and GBM. Studies have shown that mutant TP53 are major determinants of the tumor immune composition by inducing genomic instability^28^. The above results strongly indicate that PILRΑ may be implicated in regulating cancer-related immune processes, thereby potentially playing a role in immunotherapy.

The presence of certain immune cells, particularly T cells, within the tumor microenvironment has been associated with an increased likelihood of durable response to immunotherapies^29^. Therefore, we further explored the relationship between PILRΑ expression and cancer immunotherapies. Currently, multiple predictive biomarkers for immunotherapies have emerged, such as NEO, TMB, MSI and MMR^30, 31^. Tumor neoantigens, which are mainly generated due to genomic instability, are tumor-specific antigens that predicted improved responses to immunotherapy in various cancers^32, 33^. By analyzing the correlation between PILRΑ expression data and neoantigen, we found that PILRΑ was positively related with neoantigen in COADREAD and COAD while negatively with it in GBM, KIRC and UCS. As the main source of neoantigens, high tumor mutational burden is an important predictor of immunotherapy responses^34^. Besides, MSI and MMR could lead to accumulation of genetic mutations in cancers, for which they were included as main biomarkers for immunotherapy responses^35, 36^. Importantly, our results showed significant associations of PILRA with TMB, MSI and MMR, implying the potential of PILRΑ as a potential predictor for immunotherapy. Therefore, our study provided novel bioinformatics evidence supporting the potential involvement of PILRA in cancer immunity in various cancers.

There are some caveats to the present study. Firstly, the results concerning the oncogenic and immunologic roles of PILRΑ are achieved through bioinformatics analysis of multiple online repertoires, and therefore warrants further experimental validation to verify its cancer-related roles by performing in vitro and in vivo experiments. Besides, we only demonstrated positive correlations of PILRΑ with tumor immune infiltration, and thus the associations of PILRΑ with immune responses in various cancers need to be further explored and validated by experiments and analysis of clinical data to clarify the roles of PILRΑ in tumor-related immunity.

In summary, we conducted integrative bioinformatics analysis of the expression profiles and immunological roles of PILRΑ in pan-cancer. Our results demonstrated that PILRΑ was dysregulated in various cancers and positively mediated tumor immune infiltration, which provides a novel insight into the potential mechanism underlying the modulation of the immune landscape within tumor microenvironment. In conclusion, we unveiled that PILRΑ was implicated in immune responses and cancer-related immunity.

## Materials and methods

### Data acquisition and processing

The genomic and epigenomic data of PILRΑ and related clinical information of 33 common cancer types were downloaded from the Cancer Genome Atlas (TCGA, https://portal.gdc.cancer.gov/). Meanwhile, publicly available PILRΑ gene expression data of normal tissues were acquired from genotype-tissue expression database (GTEx, http://commonfund.nih.gov/GTEx/). The comparisons between cancerous and adjacent normal tissues were carried out using both TCGA and GTEx datasets. To normalize the gene expression among different samples, gene expression levels were universally presented as transcripts per million (TPM).

### Clinical samples

The tissue samples used in this study were obtained from NSCLC cancer patients with no history of radiotherapy or chemotherapy at People’s Hospital of Deyang City. The investigation was approved by the ethics committee of People’s Hospital of Deyang City.

### Comparison of differential expression of PILRΑ between cancerous and normal tissues

The PILRΑ mRNA expression differences between cancer tissues and adjacent normal tissues were compared using TIMER2.0 website (http://timer.cistrome.org/)^37^. Furthermore, the differential PILRΑ protein expression profiles were explored through analysis of the National Cancer Institute’s Clinical Proteomic Tumor Analysis Consortium platform (CPTAC, https://proteomics.cancer.gov). The cutoff values of |Log2FC| and P-value for statistical significance were set as 1 and 0.05, respectively.

### Survival analysis and receiver operating characteristic (ROC) analysis

The prognostic value of PILRΑ, including overall survival and disease specific survival, were evaluated at a pan-cancer level by conducting univariate cox regression analysis of TCGA datasets. Besides, Kaplan–Meier (KM) curves were plotted to further confirm the relationship between PILRΑ expression and patients’ prognosis using Gene Expression Profiling Interactive Analysis 2 (GEPIA2.0, http://gepia2.cancer-pku.cn)^38^, PrognoScan (http://www.prognoscan. org/)^39^ and Kaplan–Meier plotter online database (kmplot.com/)^40^. The log-rank *p* value and hazard ratio (HR) were calculated to determine the prognostic value of PILRΑ. A *p* value of smaller than 0.05 was considered to be of statistical significance.

The diagnostic potential of PILRΑ in pan-cancer was assessed utilizing area under the ROC curves (AUC) based on TCGA datasets. Specifically, AUC > 0.9 corresponds to a good level of prediction accuracy while 0.9 > AUC > 0.7 is linked to a moderate level of prediction accuracy.

### Analysis of the genomic alteration of PILRΑ in pan-cancer

The pan-cancer analysis was carried out on the PILRΑ genomic alteration landscape, comprising mutation, amplification and deep deletion, using the Cancer Types Summary module of the cBioPortal online web tool (https://www.cBioPortal.org/)^41^. The mutation landscape of PILRΑ protein was analyzed and displayed by combining the processed SNV data with the protein domains derived from the “maftools” R package in pan-cancer.

### Pan-cancer analysis of the immunological roles of PILRΑ

To investigate the roles of PILRΑ expression in immune infiltration, the stromal, immune, and ESTIMATE scores of each tumor sample were calculated based on PILRΑ gene expression via R package “ESTIMATE” (version 1.0.13) (https://bioinformatics.mdanderson.org/)^42^. Next, the Pearson’s correlation coefficient of PILRΑ expression and immune infiltration within tumor microenvironment (TME) in pan-cancer was calculated using the R package psych (version 2.1.6). In addition, the separate correlation between PILRΑ and infiltration of B cell, T cell CD4, T cell CD8, Neutrophil, Macrophage and dendritic cell (DC) was evaluated using TIMER online tool of the R package “IOBR” (version 0.99.9) (http://timer.comp-genomics.org/)^43^.

### Investigation of the correlation of PILRΑ with tumor antigen and epigenetic modulation

The effects of PILRΑ in anti-cancer immunity were analyzed based on TCGA datasets. A total of 47 immune checkpoint (ICI) genes were retrieved, and the expression correlation between these genes and PILRΑ was calculated and displayed in the fashion of a heatmap. Spearman’s correlation coefficient for ranked data was calculated to assess the association between PILRΑ gene expression and microsatellite instability (MSI) and tumor mutation burden (TMB) of each tumor sample. Additionally, Pearson’s correlation coefficient was employed to present the relationship between PILRΑ expression and the number of neo antigen count (NEO). *P* < 0.05 indicates statistical significance.

The correlation between PILRΑ and 44 RNA modulating genes including N1‐methyladenosine (m1A), 5‐methylcytosine (m5C), and N6‐methyladenosine (m6A) modification genes was presented in a heatmap based on UCSC database (https://xenabrowser.net/)^44^. In addition, the correlation between PILRΑ and DNA methyltransferase (including DNMT1, DNMT2, DNMT3A, and DNMT3) were analyzed.

### Enrichment analysis

To observe the regulatory mechanisms of PILRΑ in tumor cells, tumor samples were divided into two groups according to the high and low expression of PILRΑ. Gene Sets Enrichment Analysis (GSEA) analysis on PILRΑ was conducted to enrich and visualize related gene ontology (GO) and Kyoto Encyclopedia of Genes and Genomes (KEGG) pathway. |NES|> 1 and FDR < 0.05 were set as cutoffs for statistical significance. Besides, the STRING platform (https://string-db.org) was used to plot the protein–protein interaction (PPI) network diagrams for PILRΑ^45^.

### Immunohistochemistry (IHC) staining

To verify the protein expression of PILRA in cancer tissues relative to that in normal ones, IHC staining was performed according to the manufacturer’s protocols. Four pairs of Formalin-Fixed Paraffin-Embedded (FFPE) tissue sections (cancer and adjacent normal tissues) of NSCLC patients, were sampled. The slides were first subjected to dewaxing, hydration, incubation with 3% hydrogen peroxide and antigen retrieval (AR) by heating. Next, the tissues were successively incubated with primary antibody against PILRA (1:100, abcam) at 4 °C overnight and secondary antibody for 1 h at room temperature. Then, the tissues were stained with 3,3’-diaminobenzidine (DAB) and counterstained by hematoxylin. Images were taken under using eclipse 80i (Nikon, Japan) magnified  200×.

### Statistical analysis

The differences of gene expression between independent groups were compared using Student’s t-test. The statistical significance of survival analysis was assessed by conducting a log rank test. The Spearman’s and Pearson’s correlation coefficients were applied to quantify and estimate the correlation between PILRΑ and other variables. *P*-value less than 0.05 was considered statistically significant.

### Ethics declarations

All experiments involving human participants were performed in accordance with the guidelines and regulations of the ethics committee of People’s Hospital of Deyang City (Declaration of Helsinki) (Approval number: 2022-04-102-K01). The need for informed consent was waived by the ethics committee of People’s Hospital of Deyang City owing to the retrospective nature of the study.

### Supplementary Information

Supplementary Figures.

## Data Availability

The datasets used in this study can be found in online repositories, the name of which could be found in the article.

## Abbreviations

PILRA: Paired immunoglobulin-like type 2 receptor alpha
ITIMs: Immunoreceptor tyrosine-based inhibition motifs
BRCA: Breast invasive carcinoma
CHOL: Cholangiocarcinoma
ESCA: Esophageal carcinoma
GBM: Glioblastoma multiforme
HNSC: Head and neck squamous cell carcinoma
KIRC: Kidney renal clear cell carcinoma
KIRP: Kidney renal papillary cell carcinoma
STAD: Stomach adenocarcinoma
THCA: Thyroid carcinoma
UCEC: Uterine corpus endometrial carcinoma
LUAD: Lung adenocarcinoma
LUSC: Lung squamous cell carcinoma
PAAD: Pancreatic adenocarcinoma
LAML: Acute myeloid leukemia
DLBC: Diffuse large B-cell lymphoma
THYM: Thymoma
HCC: Hepatic cell carcinoma
OS: Overall survival
DSS: Disease specific survival
GBMLGG: Glioma
LGG: Brain lower grade glioma
TGCT: Testicular germ cell tumors
SKCM: Skin cutaneous melanoma
CESC: Cervical squamous cell carcinoma and endocervical adenocarcinoma
UVM: Uveal melanoma
OV: Ovarian serous cystadenocarcinoma
SARC: Sarcoma
ACC: Adrenocortical carcinoma
UCS: Uterine carcinosarcoma
READ: Rectal cancer
COADREAD: Colon adenocarcinoma/rectum adenocarcinoma esophageal carcinoma
COAD: Colon adenocarcinoma
ICGs: Immune check point genes
MMR: Mismatch repair
TMB: Tumor mutation burden
MSI: Microsatellite instability
NEO: Neoantigen
NF1: Neurofibromatosis 1
