# Supplementary material for: Comprehensive analysis of PILRΑ’s association with the prognosis, tumor immune infiltration, and immunotherapy in pan-cancer
Source: Sci Rep. 2023 Aug 31;13:14334. doi: 10.1038/s41598-023-41649-6 (PMC10471747; doi:10.1038/s41598-023-41649-6)
Supplement: Supplementary file 1 — Supplementary Figures. [file 41598_2023_41649_MOESM1_ESM.pdf]

Comprehensive Analysis of PILRA's Association with the Prognosis, Tumor Immune Infiltration, and Immunotherapy in Pan-cancer

Qiao Li [1], Zhirong Yang [1], Xiaoyan He [1], Xin Yang [2*]

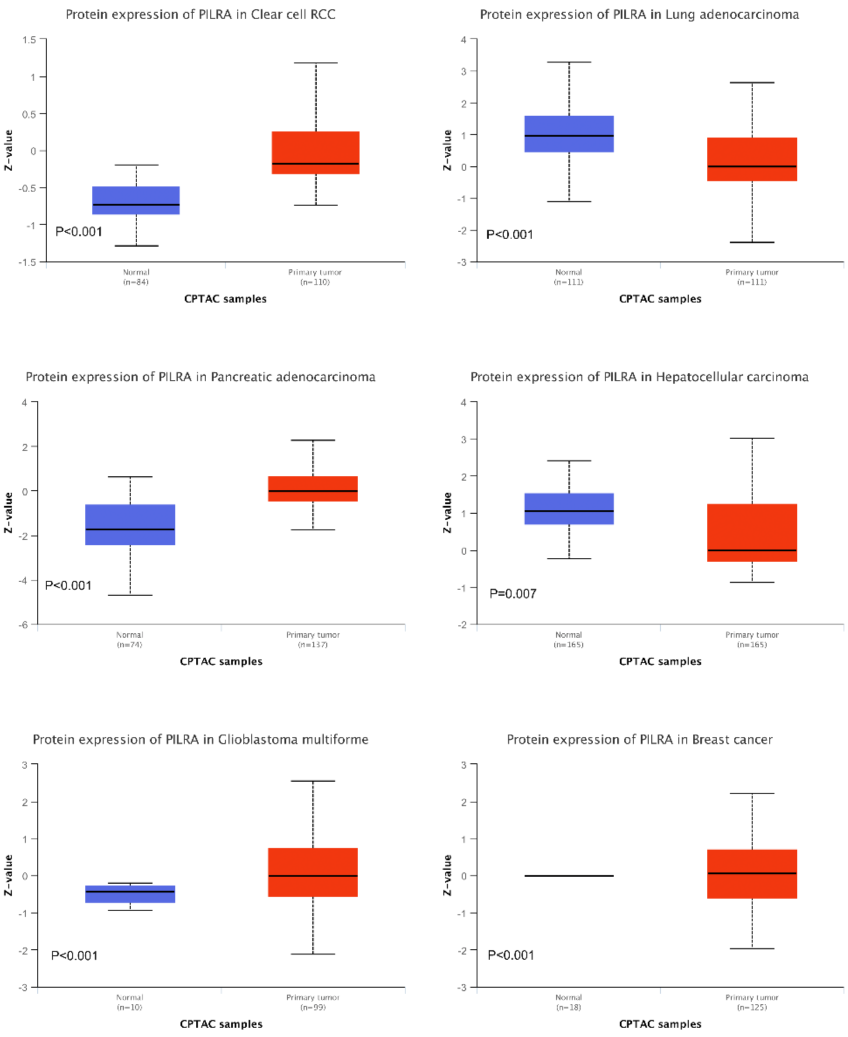

Supplementary figure 1 | The expression differences of PILRA at protein levels were compared using CPTAC in pan-cancer, Related to Figure 1.

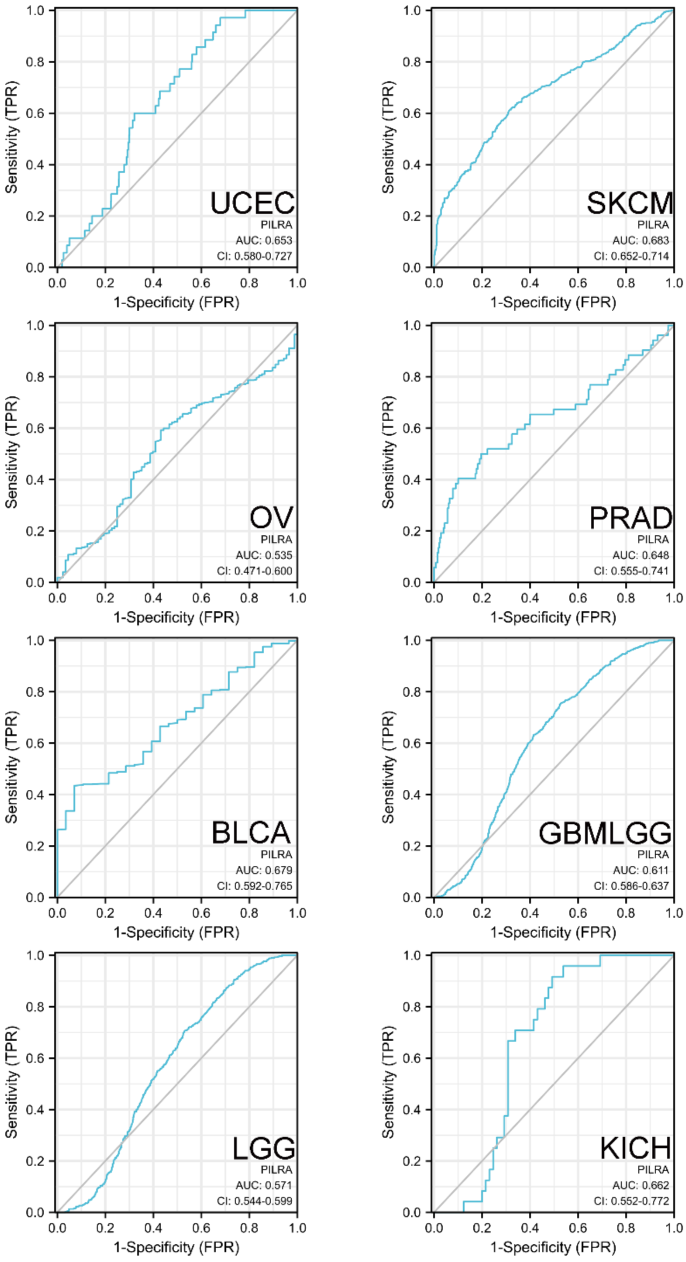

Supplementary figure 2 | ROC curve analysis evaluating the diagnostic potency of PILRA in pan-cancer based on TCGA datasets, Related to Figure 3. 8 cancers with AUC < 0.7 for PILRA were displayed here. $P < 0.05$ was considered significant.
